# Supplementary material for: Objective sleep quality predicts subjective sleep ratings
Source: Sci Rep. 2024 Mar 11;14:5943. doi: 10.1038/s41598-024-56668-0 (PMC10928218; doi:10.1038/s41598-024-56668-0)
Supplement: Supplementary file 1 — Supplementary Information. [file 41598_2024_56668_MOESM1_ESM.docx]

Supplementary tables

|  | Model 1 | | Model 2 | | Model 3 | | Model 4 | |
| --- | --- | --- | --- | --- | --- | --- | --- | --- |
|  | OR | p | OR | p | OR | p | OR | p |
| Sleep Efficiency | 1.132 | <0.001 |  |  |  |  |  |  |
| TST |  |  | 0.977 | <0.001 | 0.985 | 0.004 | 0.979 | 0.001 |
| SOL |  |  | 1.004 | <0.001 | 1.005 | <0.001 | 1.005 | 0.004 |
| WASO |  |  | 0.984 | 0.006 | 0.985 | 0.020 | 0.983 | 0.016 |
| REM % |  |  |  |  | 1.156 | 0.012 | 1.157 | 0.028 |
| REM Latency |  |  |  |  | 0.995 | 0.030 | 0.994 | 0.034 |
| N3 % |  |  |  |  | 1.223 | 0.001 | 1.219 | 0.004 |
| N3 Latency |  |  |  |  | 1.003 | 0.615 | 1.007 | 0.287 |
| N2 % |  |  |  |  | 1.162 | 0.011 | 1.144 | 0.049 |
| N2 Latency |  |  |  |  | 0.990 | 0.553 | 0.965 | 0.072 |
| Awakenings |  |  |  |  | 0.991 | 0.588 | 0.989 | 0.598 |
| Delta Power |  |  |  |  |  |  | 0.513 | 0.486 |
| Sigma Power |  |  |  |  |  |  | 2.676 | 0.444 |
|  | Number of participants | 246 | Number of participants | 246 | Number of participants | 246 | Number of participants | 235 |
|  | Number of nights | 1375 | Number of nights | 1375 | Number of nights | 1364 | Number of nights | 1141 |
|  | Incremental R-squared | 0.33 | Incremental R-squared | 0.11 | Incremental R-squared | 0.11 | Incremental R-squared | 0.13 |

**Supplementary Table S1.** Within-participant effects on subjectively rated deep sleep. The table contains fixed effects associated with the deviation of objective sleep quality metrics from individual means. The table presents odds ratios, indicating the impact of a one-unit change in objective sleep quality metrics on the odds of a response indicating deep sleep based on first GSQS item. Sleep metrics are expressed as percentage points for sleep efficiency and sleep composition, minutes for total sleep time and sleep latency, total number for awakenings and log10 microvolt/sec^2^ for relative power. Incremental R^2^ refers to the variance accounted for by the models in addition to the variance accounted for by the random intercept and control variables. R^2^ values are shown for the full model, not only within-individual effects.

|  |  | Model 1 |  |  | Model 2 |  |  | Model 3 |  |  | Model 4 |  |
| --- | --- | --- | --- | --- | --- | --- | --- | --- | --- | --- | --- | --- |
|  | B | SE | p | B | SE | p | B | SE | p | B | SE | p |
| Sleep Efficiency | -0.051 | 0.012 | <0.001 |  |  |  |  |  |  |  |  |  |
| TST |  |  |  | 0.009 | 0.004 | 0.013 | 0.009 | 0.004 | 0.031 | 0.009 | 0.005 | 0.073 |
| SOL |  |  |  | -0.004 | 0.001 | <0.001 | -0.003 | 0.001 | 0.009 | -0.004 | 0.001 | 0.004 |
| WASO |  |  |  | 0.005 | 0.005 | 0.340 | 0.005 | 0.005 | 0.367 | 0.008 | 0.006 | 0.196 |
| REM % |  |  |  |  |  |  | -0.025 | 0.047 | 0.599 | -0.002 | 0.052 | 0.973 |
| REM Latency |  |  |  |  |  |  | 0.003 | 0.002 | 0.135 | 0.002 | 0.002 | 0.381 |
| N3 % |  |  |  |  |  |  | -0.016 | 0.048 | 0.741 | 0.003 | 0.053 | 0.952 |
| N3 Latency |  |  |  |  |  |  | -0.003 | 0.004 | 0.339 | 0.003 | 0.005 | 0.596 |
| N2 % |  |  |  |  |  |  | -0.021 | 0.048 | 0.669 | 0.010 | 0.054 | 0.849 |
| N2 Latency |  |  |  |  |  |  | 0.008 | 0.013 | 0.536 | 0.018 | 0.016 | 0.271 |
| Awakenings |  |  |  |  |  |  | -0.002 | 0.014 | 0.898 | -0.001 | 0.016 | 0.958 |
| Delta Power |  |  |  |  |  |  |  |  |  | 0.367 | 0.707 | 0.604 |
| Sigma Power |  |  |  |  |  |  |  |  |  | 0.818 | 0.934 | 0.381 |
|  | Number of participants | | 246 | Number of participants | | 246 | Number of participants | | 246 | Number of participants | | 235 |
|  | Number of nights | | 1376 | Number of nights | | 1376 | Number of nights | | 1365 | Number of nights | | 1139 |
|  | Incremental R-squared | | 0.01 | Incremental R-squared | | 0.02 | Incremental R-squared | | 0.01 | Incremental R-squared | | 0.04 |

**Supplementary Table S2.** Within-participant effects on the subjective level of restedness. The table contains fixed effects associated with the deviation of objective sleep quality metrics from individual means. The table contains unstandardized regression coefficients, showing the expected change in restedness (in Likert points) as a function of a one-unit increase in sleep metrics. Sleep metrics are expressed as percentage points for sleep efficiency and sleep composition, minutes for total sleep time and sleep latency, total number for awakenings and log10 microvolt/sec^2^ for relative power. Incremental R^2^ refers to the variance accounted for by the models in addition to the variance accounted for by the random intercept and control variables. R^2^ values are shown for the full model, not only within-individual effects.

|  | Model 1 | | Model 2 | | Model 3 | | Model 4 | |
| --- | --- | --- | --- | --- | --- | --- | --- | --- |
|  | OR | p | OR | p | OR | p | OR | p |
| Sleep Efficiency | 1.080 | <0.001 |  |  |  |  |  |  |
| TST |  |  | 0.977 | <0.001 | 0.980 | 0.030 | 0.980 | 0.084 |
| SOL |  |  | 1.001 | 0.458 | 1.001 | 0.794 | 0.999 | 0.717 |
| WASO |  |  | 0.994 | 0.503 | 0.997 | 0.735 | 0.998 | 0.886 |
| REM % |  |  |  |  | 1.110 | 0.222 | 1.165 | 0.135 |
| REM Latency |  |  |  |  | 1.002 | 0.572 | 1.006 | 0.248 |
| N3 % |  |  |  |  | 1.099 | 0.266 | 1.134 | 0.209 |
| N3 Latency |  |  |  |  | 0.996 | 0.762 | 0.998 | 0.896 |
| N2 % |  |  |  |  | 1.080 | 0.380 | 1.128 | 0.244 |
| N2 Latency |  |  |  |  | 1.035 | 0.398 | 1.024 | 0.604 |
| Awakenings |  |  |  |  | 1.015 | 0.539 | 1.028 | 0.330 |
| Delta Power |  |  |  |  |  |  | 3.024 | 0.211 |
| Sigma Power |  |  |  |  |  |  | 0.208 | 0.084 |

**Supplementary Table S3.** Between-participant effects on subjectively rated deep sleep. The table contains fixed effects associated with the individual means of subjective sleep quality. The table presents odds ratios, indicating the impact of a one-unit change of sleep metrics on the odds of a response indicating poor sleep based on first GSQS item. Sleep metrics are expressed as percentage points for sleep efficiency and sleep composition, minutes for total sleep time and sleep latency, total number for awakenings and log10 microvolt/sec^2^ for relative power.

|  |  | Model 1 |  |  | Model 2 |  |  | Model 3 |  |  | Model 4 |  |
| --- | --- | --- | --- | --- | --- | --- | --- | --- | --- | --- | --- | --- |
|  | B | SE | p | B | SE | p | B | SE | p | B | SE | p |
| Sleep Efficiency | -0.034 | 0.020 | 0.085 |  |  |  |  |  |  |  |  |  |
| TST |  |  |  | 0.008 | 0.007 | 0.243 | 0.012 | 0.009 | 0.189 | 0.012 | 0.011 | 0.275 |
| SOL |  |  |  | 0.000 | 0.002 | 0.860 | 0.001 | 0.002 | 0.713 | 0.000 | 0.002 | 0.928 |
| WASO |  |  |  | 0.003 | 0.010 | 0.797 | -0.001 | 0.010 | 0.946 | -0.003 | 0.011 | 0.802 |
| REM % |  |  |  |  |  |  | 0.015 | 0.085 | 0.858 | 0.015 | 0.095 | 0.876 |
| REM Latency |  |  |  |  |  |  | -0.005 | 0.004 | 0.247 | -0.005 | 0.005 | 0.312 |
| N3 % |  |  |  |  |  |  | 0.034 | 0.084 | 0.686 | 0.045 | 0.093 | 0.628 |
| N3 Latency |  |  |  |  |  |  | 0.006 | 0.012 | 0.636 | 0.010 | 0.014 | 0.470 |
| N2 % |  |  |  |  |  |  | 0.029 | 0.086 | 0.734 | 0.044 | 0.096 | 0.647 |
| N2 Latency |  |  |  |  |  |  | 0.027 | 0.039 | 0.498 | 0.025 | 0.042 | 0.559 |
| Awakenings |  |  |  |  |  |  | -0.004 | 0.025 | 0.879 | 0.003 | 0.027 | 0.918 |
| Delta Power |  |  |  |  |  |  |  |  |  | 0.661 | 0.848 | 0.436 |
| Sigma Power |  |  |  |  |  |  |  |  |  | -1.194 | 0.874 | 0.172 |

**Supplementary Table S4.** Between-participant effects on the subjective level of restedness. The table contains fixed effects associated with the individual means of objective sleep quality, regressed on subjective rated restedness. The table contains unstandardized regression coefficients, showing the expected change in the level of restedness (in Likert points) as a function of a one-unit increase in sleep metrics. Sleep metrics are expressed as percentage points for sleep efficiency and sleep composition, minutes for total sleep time and sleep latency, total number for awakenings and log10 microvolt/sec^2^ for relative power.

|  | **Adjusted** | | **Raw** | |
| --- | --- | --- | --- | --- |
|  | Within-only model | Full model | Within-only model | Full model |
| Model 1 | 0.154 | 0.159 | 0.154 | 0.160 |
| Model 2 | 0.186 | 0.190 | 0.187 | 0.192 |
| Model 3 | 0.197 | 0.197 | 0.202 | 0.207 |
| Model 4 | 0.167 | 0.173 | 0.175 | 0.189 |

**Supplementary Table S5.** Incremental R^2^ values (adjusted for degrees of freedom, as in Table 3, or raw) from models using only within-participant effects. Values from the full models also including between-participant effects, as reported in the main manuscript, are shown for comparison.

| Within Subject | | | | |  | Between Subject | | | | |
| --- | --- | --- | --- | --- | --- | --- | --- | --- | --- | --- |
| Variable | Original | GESD | Winsorized | Gamma regression |  | Variable | Original | GESD | Winsorized | Gamma regression |
| SOL | 0,029*** | 0,046*** | 0,039*** | -0,001*** |  | SOL | 0,024 | 0,034* | 0,041** | -0,001 |
| WASO | 0,04*** | 0,056*** | 0,053*** | -0,001*** |  | WASO | 0,04*** | 0,054*** | 0,045* | -0,001 |
| TST | -0,012*** | -0,011*** | -0,012*** | 0,001*** |  | TST | -0,006* | -0,008** | -0,007* | <0,001 |
| N2% | -0,199** | -0,232*** | -0,176** | 0,003 |  | N2% | -0,059 | 0,030 | -0,084 | <0,001 |
| N3% | -0,232*** | -0,266*** | -0,207** | 0,005 |  | N3% | -0,056 | 0,033 | -0,068 | <0,001 |
| REM% | -0,215** | -0,244*** | -0,192** | 0,005 |  | REM% | -0,059 | 0,024 | -0,081 | 0,001 |
| N2 latency | 0,035* | 0,032 | 0,049* | -0,001** |  | N2 latency | -0,036 | -0,005 | -0,050 | 0,002 |
| N3 latency | 0,005 | -0,001 | 0,005 | <0,001 |  | N3 latency | 0,003 | 0,008 | 0,017 | <0,001 |
| REM latency | 0,005 | 0,004 | 0,005 | <0,001 |  | REM latency | <0,001 | -0,001 | 0,001 | <0,001 |
| Awakenings | -0,033 | -0,053* | -0,045* | -0,001 |  | Awakenings | -0,005 | -0,001 | 0,008 | -0,001 |

**Supplementary Table S6.** The associations between subjective and objective sleep quality as derived from Model 3. We show the original results side by side those obtained using three different outlier exclusion methods: Generalized Extreme Studentized Deviates (GESD), winsorization, and a gamma distribution with an inverse link function. Regression coefficients can be directly compared across the first three methods, but not with gamma regression where the data is transformed. Note that the significance of the original estimates is preserved across most methods, especially for the more important within-subject results. (* p<0.05, ** p<0.01**, *** p<0.001)

Supplementary figures


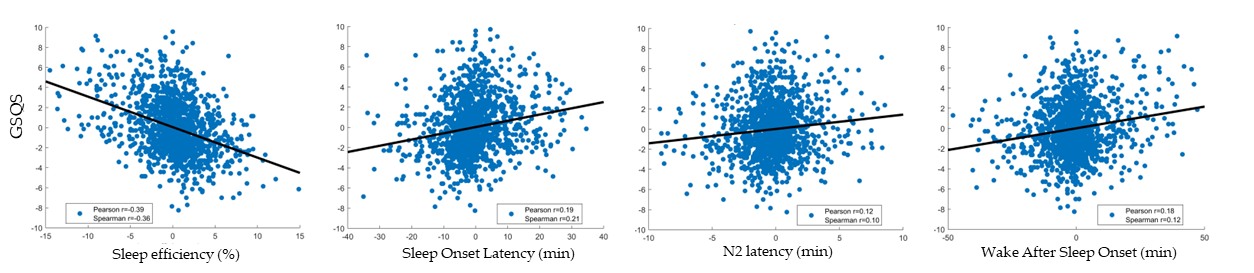


**Supplementary Figure S1.** Within-participant associations between indicators of subjective sleep quality (GSQS total score, vertical axis) and objective sleep metrics (separate panels, horizontal axis) after outlier elimination using the Generalized Extreme Studentized Deviate (GESD) method. The scatterplots show deviations from the individual means, pooled across participants. Because the plots display raw residuals, data points are centered around zero as described in the manuscript. Sleep metrics are selectively presented if they underwent outlier elimination.


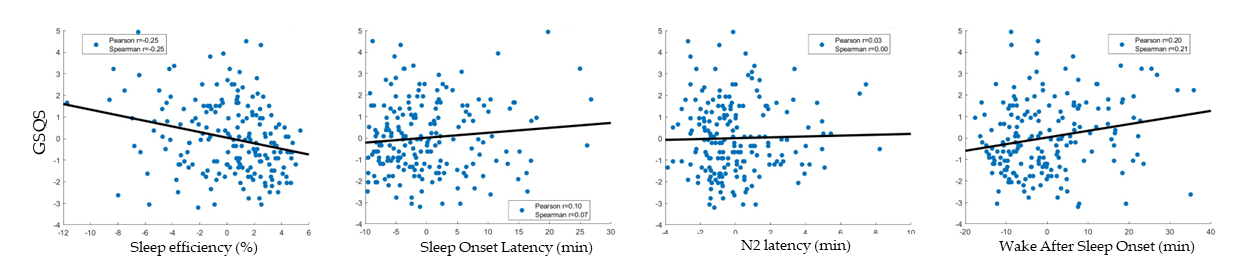


**Supplementary Figure S2.** Between-participant associations between indicators of subjective sleep quality (GSQS total score, vertical axis) and objective sleep metrics (separate panels, horizontal axis) after outlier elimination using the Generalized Extreme Studentized Deviate (GESD) method. The scatterplots show individual means. Because the plots show raw residuals, data points are centered around 0 as described in the manuscript. Sleep metrics are selectively presented if they underwent outlier elimination.
